# Ureter Injury in Total Laparoscopic Hysterectomy

**DOI:** 10.1155/2023/5071080

**Published:** 2023-08-18

**Authors:** Hiroharu Kobayashi, Aimi Oda, Yoshihiko Matsuzaki, Yuki Kondo, Yuri Hamada, Masaru Nagashima, Misa Kobayashi, Yoshihiro Takaki, Hiroshi Adachi

**Affiliations:** Department of Gynecology, Seirei Hamamatsu General Hospital, Hamamatsu, Japan

## Abstract

**Objective:**

To identify surgical manipulations that caused ureter injury during total laparoscopic hysterectomy (TLH) and evaluate the surgical manipulations to identify ways to prevent such injury. *Patients and Methods*. This single-center, cross-sectional study included 1135 cases of TLH performed for benign diseases from January 2009 to December 2021. Seven cases (0.6%) that needed ureteral stent placement intra- or postoperatively for ureter injury were included. We identified the surgical manipulations that caused ureter injury from surgical videos.

**Results:**

Two cases had adhesions around the bladder pillar, and the ureter sustained a thermal injury during the cardinal ligament transection. One case had severe endometriosis, and the ureter was bluntly damaged when the adhesion was released. In one case, the ureter was thermally damaged during bipolar hemostasis for uterine artery bleeding. In two cases, the obliterated umbilical artery was mistaken for the ureter, and the real ureter was injured. In one case, ureteral peristalsis was inhibited by a pelvic abscess caused by postoperative infection.

**Conclusion:**

To prevent ureter injury during TLH, the ureter should be isolated in case of severe adhesion. Moreover, the following could be considered: (1) expand Okabayashi's pararectal space lateral to the uterosacral ligament, (2) perform dissection sharply using a monopolar or scissors forceps when releasing adhesion, (3) clarify the anatomy around the ureter for cases needing hemostasis, (4) repeatedly confirm the ureter with its peristalsis even after its isolation, (5) for severe adhesion cases, reduce infection risk by drain placement and administering antibiotics, and (6) use a delineator cup.

## 1. Introduction

Ureter injury is a complication that must be avoided in total hysterectomy. It has been reported that laparoscopic surgery has a higher frequency of ureter injury than open surgery in total hysterectomy (laparoscopy: 0.13% and open surgery: 0.04%) [1]. We extracted the cases of ureter injury from a total laparoscopic hysterectomy (TLH) performed for benign diseases in our hospital and identified the surgical manipulations that have caused ureter injury from surgical videos. We examined surgical manipulations to avoid ureter injury in TLH.

## 2. Materials and Methods

Of 1135 cases of TLH performed for benign diseases in our hospital from January 2009 to December 2021, 7 cases (0.6%) needed ureteral stent placement during or after surgery for ureter injury. From surgical videos of these seven cases, we identified surgical manipulations that caused ureteral damage and examined surgical manipulations to prevent ureter injury.

The surgical method of TLH in our hospital is described below. The patient is fixed at a lithotomy position with a low head of 20°–30°. A manipulator with a delineator cup (KOH cup) is transvaginally placed into the uterus. If the manipulator with a KOH cup cannot be attached due to a narrow vagina and narrow external uterine ostium, a manipulator without a KOH cup is inserted into the uterus. Trocars are located in a modified diamond shape. Moreover, 12 mm-diameter and 75 mm-long Excel trocars are inserted into the navel by the semi-open method. Subsequently, 5 mm-diameter and 70 mm-long EZ trocars are inserted into the left, middle, and right of the lower abdomen. Pneumoperitoneum pressure is maintained at 8 mmHg. The camera is a 30° oblique-viewing endoscope of 5 mm diameter. Probe Plus II, which is a monopolar electric device with a suction function, Olympus bipolar and Enseal as a thermal sealing device are used. The vesicouterine peritoneum is incised between the bilateral round ligaments. The round ligament is transected, the retroperitoneal space is expanded, and the ureter and uterine artery are identified. The uterine artery is isolated and transected. The bladder is detached from the cervical and vaginal anterior wall and is moved caudally. The ovarian ligaments are transected if the ovaries are preserved, or the suspensory ligaments are transected if the ovaries are removed. The posterior uterine broad ligaments are incised along the uterus to the uterosacral ligaments, paying attention to the running of the ureter. Okabayashi's pararectal space lateral to the uterosacral ligament is expanded, and the ureter is moved caudally. The uterosacral ligament is transected. The cardinal ligaments, which include the ascending branch of the uterine arteries and veins, are transected with Enseal. The vaginal wall is incised with a monopolar electric device all around along the edge of the KOH cup that fits into the vaginal fornix. If the KOH cup has not been attached, the manipulator is removed, and a vaginal pipe is inserted into the vagina. The vaginal wall is incised along the edge of the vaginal pipe that fits into the vaginal fornix. The uterus is transvaginally morcellated and collected. The vaginal stump is sutured with a single nodule of 1PDS Plus. The peritoneum is continuously sutured with a 2-0 PDS to cover the vaginal stump. Diluted indigo carmine is intravenously administered, and urine outflow from bilateral ureteral orifices is confirmed with cystoscopy (30° oblique-viewing endoscope of 4 mm diameter). Interseed is placed around the vaginal stump, and the drain is placed in the cul-de-sac.

Written informed consent that the surgical video may be used anonymously for clinical research was obtained preoperatively from the patients. This study was approved by our Institutional Review Board on June 16, 2021 (research approval number 3637).

## 3. Results

Table 1 describes surgical indication, intraabdominal adhesion, the weight of the uterus, the injury side, vaginal instruments, and the mechanism of ureter injury in each case. The surgical indication of six cases was myoma, and that of one case was adenomyosis. There were five cases with strong adhesion due to endometriosis or a history of surgery, such as myomectomy or cesarean section, and two cases with no adhesion. In one case, the weight of the uterus was more than 1 kg, but in all others, it was less than 500 g. Four cases had right injuries, two cases had left injuries, and one case had bilateral injuries. Intravaginal instruments were vaginal pipe in five cases, and delineator cup in two cases.

**Figure 1:**
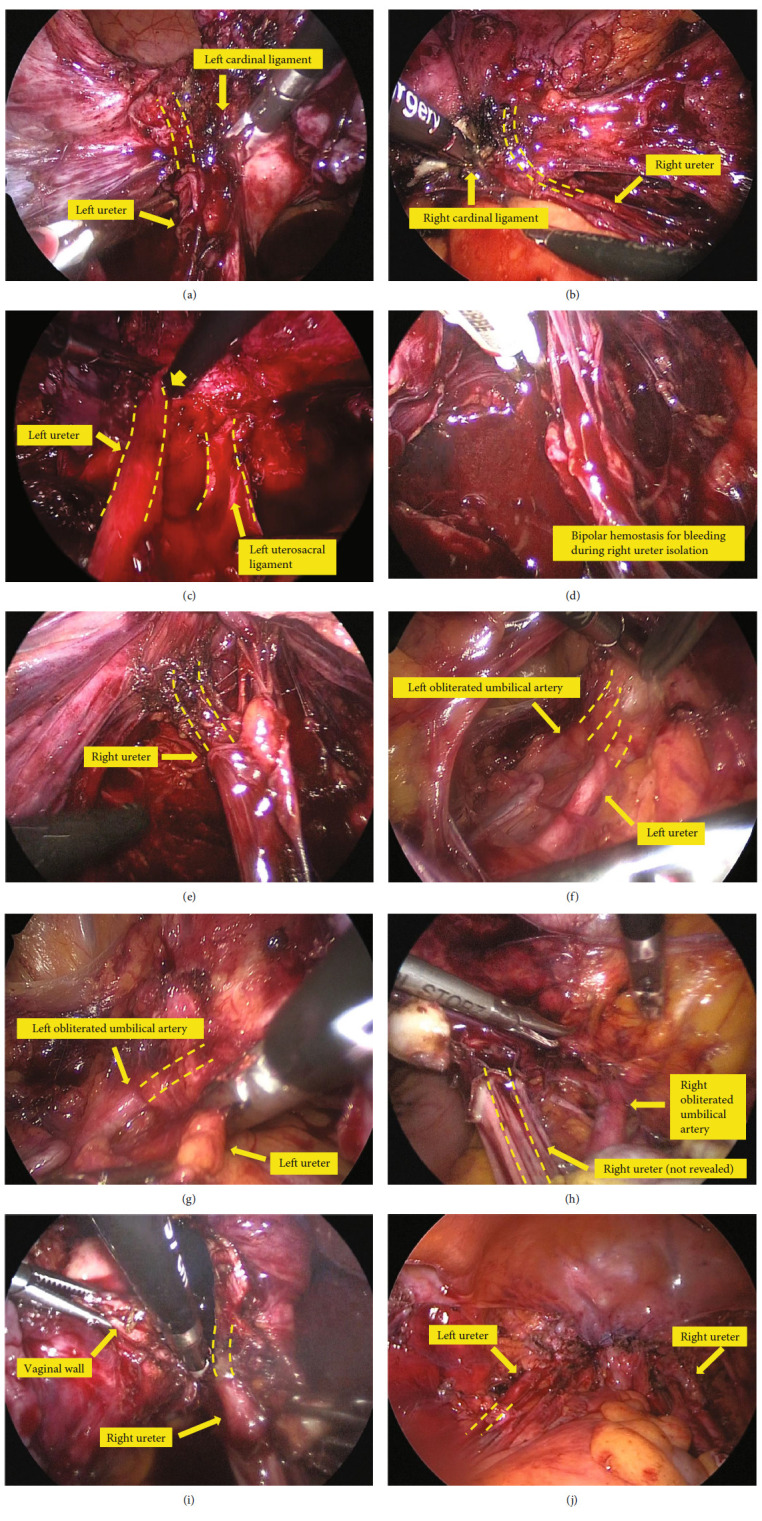
Still images of the intraoperative videos (the cases of ureter injury). (a) When the left cardinal ligament was transected, the left ureter, which run in close proximity, was thermally injured. (b) When the right cardinal ligament was transected, the right ureter, which run in close proximity, was thermally injured. (c) The left ureter was injured by a blunt manipulation when separating the left ureter from the left uterosacral ligament. (d) The right ureter was thermally injured while attempting to stop bleeding from the right uterine artery. (e) The right ureter, which is thought to have been thermally damaged, is shown in the figure. (f) The left ureter and the left obliterated umbilical artery running lateral to it were identified. (g) Afterwards, the left obliterated umbilical artery was misidentified as the left ureter, which was severed. (h) The right obliterated umbilical artery was mistaken for the right ureter. (i) When the vaginal wall was incised, the adjacent right ureter was thermally damaged with a thermal sealing device.(j) A pelvic abscess caused by postoperative infection inhibited ureteral peristalsis, resulting in bilateral hydronephrosis.

Case 1 had a history of cesarean section and myomectomy, which caused a vesicouterine peritoneum, especially the left bladder pillar, to be strongly adhered to. Confirming the left ureter running lateral to the left cardinal ligament, it was transected. This caused heat injury to the left ureter running nearby (Figure 1(a)). Cystoscopy after suturing the vaginal stump confirmed urine outflow from the left ureter orifice. A left ureteral stent was inserted 3 days after the surgery. Dilation of ureteral stenosis with a balloon was performed twice, and the stent was removed 10 months after the surgery.

Case 2 had a history of cesarean section and, the vesicouterine peritoneum, especially the right bladder pillar was strongly adhered. After confirming the right ureter running lateral to the right cardinal ligament, it was transected, which thermally damaged the adjacent right ureter (Figure 1(b)). Cystoscopy after suturing the vaginal stump confirmed urine outflow from the right ureteral orifice. A right ureteral stent was placed 12 days after surgery and was removed 2 months after the surgery.

In case 3 the cul-de-sac was obliterated due to endometriosis. The left ureter was injured by a blunt manipulation when separating the left ureter from the left uterosacral ligament (Figure 1(c)). Cystoscopy was not performed after suturing the vaginal stump in this case. A ureteral stent was placed 7 days after surgery and was removed 4 months after surgery.

Case 4 had a uterus weighing more than 1 kg, but there was no adhesion in the abdominal cavity. When the right uterine artery was isolated, its damaged ureteral branch bled. Its hemostasis with bipolar device thermally damaged the right ureter (Figures 1(d) and 1(e)). Cystoscopy after suturing the vaginal stump confirmed urine outflow from the right ureteral orifice. A right ureteral stent was placed 21 days after the surgery and was removed 7 months after the surgery.

Case 5 had a history of myomectomy and extensive adhesion from the bladder to the anterior wall of the uterus. The adhesion was released, the left ureter was identified, and the left uterine broad ligament was incised along the uterus. At this time, the left obliterated umbilical artery running behind the left ureter had been displaced inward due to the effect of adhesion, and this was mistaken for the previously isolated left ureter. As a result, the left ureter running in front was not recognized and transected (Figures 1(f) and 1(g)). Intravenously administered indigo carmine flowed into the abdominal cavity, which showed amputation of the left ureter. After the transition to open surgery, the left ureter was repaired, and a left ureteral stent was placed. It was removed 4 months after the surgery.

Case 6 had no adhesion in the abdominal cavity. The running of the ureters was identified, and the bilateral cardinal ligaments were transected. When the vaginal wall was incised, the adjacent right ureter was thermally damaged with a thermal sealing device. It seems that the surgeon initially correctly identified the running of the right ureter, but misidentified the right obliterated umbilical artery running outside as the right ureter at the time of the vaginal wall incision (Figures 1(h) and 1(i)). Cystoscopy after suturing the vaginal stump confirmed urine outflow from the right ureteral orifice. A right ureteral stent was placed 16 days after the surgery and was removed 2 months after the surgery.

Case 7 involved bilateral ovarian endometriotic cysts and marked adhesions around the uterus. The bilateral ureters were separated, the uterus was removed, and no obvious ureteral damage during the operation was observed (Figure 1(j)). On the 18th day after the surgery, bilateral hydronephrosis occurred from an abscess in the pelvis, which seemed to have inhibited ureteral peristalsis, and bilateral ureteral stents were placed. Five months after the surgery, the stents were removed.

## 4. Discussion

**Figure 2:**
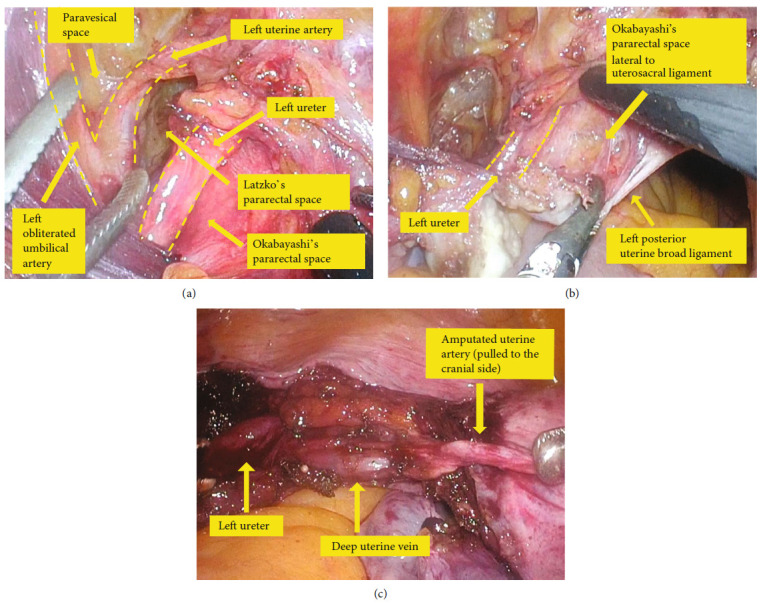
Still images of the intraoperative videos (intraoperative manipulations to prevent ureter injury in TLH). (a) The uterine artery and the ureter are naturally identified by opening three spaces (paravesical space, Latzko's pararectal space, Okabayashi's pararectal space). (b) The Okabayashi's pararectal space lateral to the uterosacral ligament is opened and the ureter is moved caudally. (c) Separation of the severed uterine artery from the ureter ensures complete safety of the ureter.

**Figure 3:**
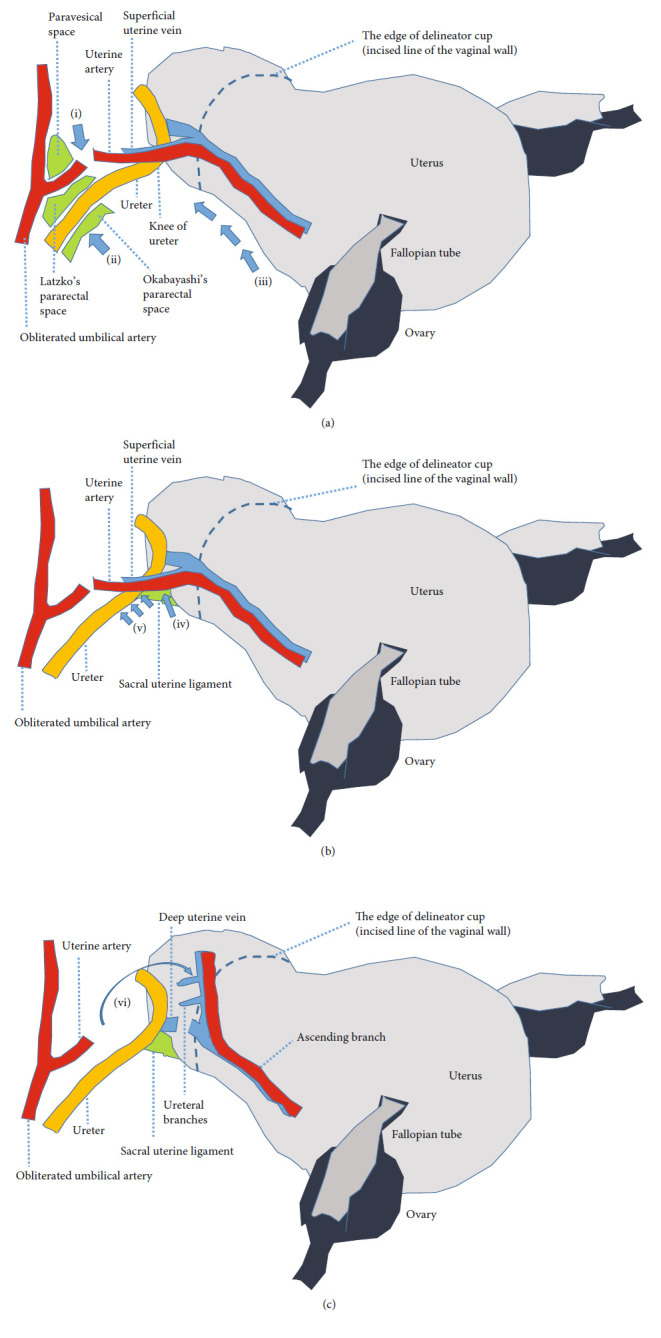
Intraoperative manipulations to prevent ureter injury in TLH. (a) (i) Separating the uterine artery away from the uterus reduces the risk of bleeding. (ii) The ureter is easier to identify by expanding Okabayashi's pararectal space. (iii) Incising the posterior uterine broad ligament closer to the uterus reduces the risk of ureteral injury. (b) (v) Okabayashi's pararectal space lateral to the uterosacral ligament is expanded to identify the ureter running caudally. (iv) The ureter is detached from the posterior uterine broad ligament and mobilized caudally. (c) (vi) Ureteral safety is fully ensured by separating the uterine artery and vein from the ureter by cutting the ureteral branches and the descending branch of the uterine artery and vein.

In cases 1 and 2, the ureter was thermally damaged during the cardinal ligament transection. In open surgery, the cardinal ligament is usually transected after ligation, so there is less risk of ureteral damage even if the ureter is close to the transected cardinal ligament. In laparoscopy, a thermal sealing device is used for cardinal ligament transection, which increases the risk of thermal damage to the adjacent ureter. Unlike ligation, a thermal sealing device may not completely stop the bleeding of transected cardinal ligament, and the hemostasis manipulation with a bipolar device further increases the risk of thermal damage to the ureter. In both cases 1 and 2, when the cardinal ligament was transected in multiple steps, the ureter attached to the posterior uterine broad ligament approached the contemplated transaction portion of the cardinal ligament, resulting in thermal damage of the ureter. This type of ureter injury can be prevented by expanding the Okabayashi's pararectal space lateral to the uterosacral ligament (Figures 2(a) and 3(b)(iv)), detaching the ureter from the posterior uterine broad ligament, and mobilizing the ureter caudally (Figure 3(b)(v)) before cardinal ligament transection. Ureter injury in case 5 could have been avoided by performing this manipulation, and it corresponds to separating the ureter from the adjacent uterosacral ligament in case of deep endometriosis, such as in case 3. If the bladder pillar adheres more strongly, the ureter is displaced inward and closer to the uterine cervix from the cranial side. In such cases, only separating the ureter from the posterior uterine broad ligament and mobilizing it caudally may not provide sufficient distance between the ureter and the transected cardinal ligament. However, it can be sufficiently taken by separating the uterine artery and vein from the ureter by cutting the ureteral branches and descending branches of the uterine artery and vein (Figures 2(c) and 3(c)(vi)). Furthermore, the transection of the anterior leaf of the vesicouterine ligament leads to a semi-radical hysterectomy. However, in benign diseases, only the above procedures may be sufficient to avoid the risk of ureter injury.

Case 3 was typical endometriosis, in which the cul-de-sac was adherently obliterated and the left ureter adhered to the left uterosacral ligament. In deep endometriosis, endometriotic focus forms around the uterosacral ligament. The obliterated umbilical artery and the ureter deviate medially, and the ureter is severely adhering to the uterosacral ligament. In such a case, the ureter must be separated from the uterosacral ligament for a complete total hysterectomy, but blunt detachment damaged the ureter. In critical situations, dissection should be done with sharp manipulation using monopolar or scissor forceps rather than by blunt manipulation. Although sharp manipulation increases the risk of bleeding, the surgeon should dissect the ureter at the very edge of it with sharp manipulation, move it outward, and stop bleeding with bipolar. If the risk of ureter injury is very high, supravaginal amputation of the uterus should be considered instead of a total hysterectomy. Nezhat and Nezhat reported hydrodissection and laser vaporization for peritoneal endometriosis, in which saline solution is injected locally into the endometriotic lesion to separate it from the surrounding tissue (ureter and blood vessels) by making a water bed subperitoneally, and vaporize the isolated peritoneal lesion using laser [2]. This method appears to be effective in preventing complications, such as ureteral injury, in the treatment of endometriosis. However, it may have limitations for deep endometriosis that forms lesions centered on the uterosacral ligament.

In case 4, when separating the uterine artery, the uterine vein or ureteral branch of the uterine artery bled, and hastened hemostasis with bipolar device caused thermal injury of the ureter running nearby. In such a case, the surgeon should not panic but rather get an assistant surgeon to press the bleeding site with forceps. Hemostasis with a bipolar device should always be performed after clarifying the anatomy around the bleeding point, identifying the ureter, and keeping a distance between the ureter and the bleeding point. The closer the uterine artery and ureter are to the uterus, the higher the risk of bleeding due to damage to the ureteral branches of the uterine artery. Therefore, the uterine artery should be separated at the outer side where the uterine artery branches off from the obliterated umbilical artery (Figure 3(a)(i)). Simultaneously, finding the uterine artery becomes easy by expanding the paravesical space inside the obliterated umbilical artery, which runs inside the external iliac vein (Figure 2(a)). Finding the ureter becomes easy by expanding the Okabayashi's pararectal space (Figure 3(a)(ii)), and this should be done outside as much as possible to reduce the risk of bleeding of the ureteral branches of the uterine artery.

In cases 5 and 6, after the ureter was identified and isolated, it was damaged because the obliterated umbilical artery running behind the ureter was mistaken for the previously isolated ureter during the subsequent manipulation. In case 5, when the left posterior uterine broad ligament was incised, the left obliterated umbilical artery was mistaken for the left ureter, and the ureter running in the front was transected. In case 6, the right obliterated umbilical artery running laterally to the right ureter was mistaken for the right ureter, which was hidden in the right posterior uterine broad ligament, and the right ureter was thermally damaged in the vaginal wall incision. In cases of severe adhesions with a history of myomectomy or cesarean section, the obliterated umbilical artery is displaced medially and sometimes runs like a ureter, and it may be mistaken for the ureter. This makes the ureter unrecognized and injured. Since the ureter is frequently peristaltic, it should always be checked for reconfirming the ureter even after isolation of the ureter. Furthermore, when incising the posterior uterine broad ligament along the uterus up to the origin of the sacral uterine ligament, the risk of ureter injury can be reduced by making the incision as close to the uterus as possible (Figure 3(a)(iii)).

In case 7, the entire pelvis adhered due to endometriosis. Small bleeding occurred in the process of releasing the adhesion, and Surgicel absorbable hemostat (Johnson and Johnson) was used to stop the bleeding. We consider that blood was accumulated in the cul-de-sac due to oozing even after the operation, and Surgicel absorbable hemostat and accumulated blood increased the risk of infection. In such cases, we should leave a drain and administer antibiotics for a few days postoperatively, which can reduce the risk of infection and abscess formation in the pelvis.

We habitually use a delineator cup attached to a manipulator in TLH for benign diseases [3]. We need to transect the cardinal ligament up to the level where the vaginal wall is incised. The delineator cup fits into the vaginal fornix, and we incise the vaginal wall marked by the edge of the delineator cup. It can prevent an unnecessary caudal transection of the cardinal ligament and resulting ureter injury. Since the ureter is never located on the cranial side of the edge of the delineator cup, it is very useful to reduce ureter injury [4]. In most cases, we use the delineator cup instead of a vaginal pipe, which is only used when the delineator cup cannot be inserted into the vagina because of a narrow vagina or narrow uterine ostium. The delineator cup was used in only two cases of the seven cases of ureter injury in this study. This suggests the usefulness of the delineator cup.

Ureter injury was reported to be more on the right side than on the left side [5]. In this study, the injury side was right in four cases of the six cases with one side injury. The operator usually stands on the left side of the patient in the diamond-shaped placement of the trocar. The right side of the uterine cervix is visually disadvantageous and forceps manipulation on the right side may be limited if the uterus is large. There was a report that the ureter intersects the cardinal ligament in a more cephalad position on the right side than on the left side [6]. The anatomical difference between the right and left running of the ureter may be one of the reasons for the bilateral difference in ureteral injury.

Most bladder injuries are found intraoperatively, but most ureter injuries are found postoperatively [5, 7, 8]. In this study as well, ureter injury was found after surgery in all cases except case 4, in which the ureter was amputated during surgery. The common symptom at the time of discovery of ureter injury was lower abdominal pain on the injured side, and common laboratory findings were a mild increase in blood creatinine and the appearance of urinary blood cells. Intraoperative cystoscopy confirmed urine outflow from the injured ureter in all of these cases which underwent cystoscopy. Therefore, routine intraoperative cystoscopy is meaningless in identifying ureter injury, especially for ureteral thermal injury [7].

Intraoperative visualization of the ureter using ureteral stent or indocyanine green has been reported as an effective way to prevent ureteral injury. Intraoperative visualization of the ureter with fluorescence using indocyanine green may be the best way to prevent ureteral injury [9]. Cases 1, 2, 4, 5, and 6 in our study could have been avoided if this method had been used. This method would be also effective for beginners of TLH. The disadvantages are the labor involved in preoperatively using a cystoscope to pour indocyanine green into the ureter and the need for a machine to detect fluorescence. With the ureteral stent in place, the ureter becomes taut, and identification of the ureter is easier. In addition, in the unlikely event of ureteral injury, subsequent repair will be easier.

In this study, five of the seven cases had strong intraabdominal adhesion. Intraabdominal adhesion due to a history of surgery or endometriosis is the strongest factor of ureter injury. Isolation of the ureter in TLH is not always a necessary manipulation in cases without intraabdominal adhesion. However, the stronger the intraabdominal adhesion, the greater the need to isolate the ureter to prevent ureter injury. To prevent ureter injury, (1) before the cardinal ligament transection, Okabayashi's pararectal space lateral to the uterosacral ligament should be expanded and the ureter should be moved to the caudal side. This corresponds to the manipulation of separating the ureter from the uterosacral ligament in a case of deep endometriosis. In a case with stronger adhesion, the ureter should be completely separated from the uterine artery and vein by cutting its descending and ureteral branches. (2) In case of severe adhesions, dissection should be performed sharply using a monopolar or scissors forceps rather than blunt manipulation. (3) The uterine artery should be isolated as close to the obliterated umbilical artery as possible because the closer it is to the uterus, the higher the risk of bleeding due to ureteral branches. If bleeding occurs, hemostasis should be performed after clarifying the anatomy around the bleeding point, identifying the ureter, and keeping a distance between the ureter and the bleeding point. (4) The ureter should be repeatedly reconfirmed by its peristalsis even after its isolation, and the posterior uterine broad ligament should be incised as close to the uterus as possible to reduce the risk of ureter injury. (5) In a case with severe adhesion, a drain tube should be placed and antibiotics administered for a while after surgery because postoperative bleeding and anti-hemostatic agent pose a risk of infection. Infection may cause abscess formation, which inhibits ureteral peristalsis and causes hydronephrosis. (6) It is recommended to use a delineator cup rather than a vaginal pipe. By doing the above procedures, ureter injury in TLH can be minimized.

This study had several limitations. There were only seven cases. The manipulations that were thought to have caused ureter injury were based on subjective considerations from the video. In some cases, the opinion of the surgeon who performed the surgery could not be obtained.

## 5. Conclusion

To prevent ureter injury during TLH, the ureter should be isolated in case of severe adhesion. Moreover, the following could be considered: (1) expand Okabayashi's pararectal space lateral to the uterosacral ligament, (2) perform dissection sharply using a monopolar or scissors forceps when releasing adhesion, (3) clarify the anatomy around the ureter for cases needing hemostasis, (4) repeatedly confirm the ureter with its peristalsis even after its isolation, (5) for severe adhesion cases, reduce infection risk by drain placement and administering antibiotics, and (6) use a delineator cup.

## Figures and Tables

**Table 1:** Characteristics of the cases.

No.	Age (years)	G	P	CS	Past history	Indications	Intra-abdominal adhesion	Operation time (min)	Bleeding (mL)	Weight of the uterus (g)	Side	Intravaginal device	Ureter injury	Stent placement
1	44	3	1	1	Myomectomy	Myoma	Strong	210	350	113	Left	Cup	Intraoperative thermal	Postoperative
2	48	1	1	1	—	Myoma	Strong	216	80	460	Right	Pipe	Intraoperative thermal	Postoperative
3	43	4	2	0	Conization	Adenomyosis	Strong	240	20	80	Left	Pipe	Intraoperative blunt	Postoperative
4	41	0	0	0	—	Myoma	No adhesion	202	140	1133	Right	Pipe	Intraoperative thermal	Postoperative
5	48	0	0	0	Myomectomy	Myoma	Strong	288	165	464	Left	Pipe	Intraoperative amputation	Intraoperative
6	49	4	2	0	—	Myoma	No adhesion	151	10	250	Right	Pipe	Intraoperative thermal	Postoperative
7	50	3	3	0	—	Myoma and endometriosis	Strong	167	315	166	Both	Cup	Postoperative infection	Postoperative

## Data Availability

The data used to support the findings of this study (still images of the intraoperative videos) are included in the article.
